# 

**Published:** 1880-03

**Authors:** 


### CONTENTS.

COMMUNICATIONS.

Extirpation of the Dental Pulp and Root Filling........................ 105
Removal of Parotid Tumor – Death from Cellulitis........................ 116
On the Value of Retaining the Roots of Teeth After the Loss of their Crowns........................ 117
A Course of Lectures on Operative Dental Surgery and Therapeutics 119
Extraction of First Molars........................ 129
Thirty-Sixth Annual Meeting—Mississippi Valley Association of Dental Surgeons........................ 133
Effects of Tobacco on the Eyes........................ 140
A New Antiseptic........................ 140
Clinical Reports........................ 142
SCIENTIFIC NEWS........................ 143
MISCELLANEOUS NOTES........................ 144
EDITORIAL........................ 147
One Step Ahead........................ 147
The Dental Student's Note Book........................ 148

